# Stability and predictability of Bayley Scales of Infant and Toddler Development: evidence from a south Indian birth cohort prospective study

**DOI:** 10.1136/bmjopen-2023-082624

**Published:** 2024-11-19

**Authors:** Beena Koshy, Manikandan Srinivasan, Rebecca Scharf, Tor A Strand, Venkata Raghava Mohan, Rachel Beulah, Sushil John, Jayaprakash Muliyil, Gagandeep Kang

**Affiliations:** 1 Christian Medical College Vellore, Vellore, Tamil Nadu, India; 2 University of Virginia, Charlottesville, Virginia, USA; 3 University of Bergen, Bergen, Norway; 4 Department of Research, Innlandet Hospital Trust, Brumunddal, Norway

**Keywords:** Developmental neurology & neurodisability, Social Cognition, Community child health

## Abstract

**Abstract:**

**Objectives:**

There is limited information about the stability and predictability of Bayley Scales of Infant and Toddler Development (BSID) assessing child development in low- and middle-income settings. The objective of the present study was to analyse stability and predictive validity of BSID using an existing birth cohort.

**Design:**

Prospective birth cohort follow-up study.

**Setting and participants:**

A community-based birth cohort of 251 newborns was recruited and followed-up in urban Vellore, South India, until 9 years of age. Using BSID-III, child development was measured at 6, 15, 24 and 36 months. Cognition was assessed using the Wechsler Preschool Primary Scales of Intelligence at 5 years, and the Malin’s Intelligence Scale for Indian Children scale at 7 and 9 years of age. The stability of BSID measurements across time points was expressed by intraclass correlation (ICC) and concordance correlation coefficients. Linear regression was used to describe the predictability of BSID-III of cognition at 5, 7 and 9 years.

**Results:**

The ICC for domain-wise BSID scores between time points of measurement suggested a weak correlation. The BSID scores at 36 months correlated best with Full-Scale Intelligence Quotient (FSIQ) at 5 years (r: 0.40–0.49), 7 years (r: 0.35–0.48) and 9 years (r: 0.36–0.38). BSID scores at 36 months predicted FSIQ better at 5, 7 and 9 years with R^2^ ranging from 23.3% to 28.6%, when compared with 24 months BSID scores (R^2^ - 16.0% to 25.9%).

**Conclusion:**

Poor stability and predictability of BSID warrant caution in the predictive projection of early childhood assessments. Better predictability of future cognition of 36 months’ BSID scores highlights its advantage over the 24 months’ assessment.

**Strengths and limitations of this study**

- This longitudinal community-based birth cohort follow-up study in a low- and middle-income settings in South India measured child development at 6, 15, 24 and 36 months and cognition at 5, 7 and 9 years of age to understand about stability and predictability of childhood development assessment tool.
- Standardised assessments with local and global quality checks were used and the birth cohort had good data granularity in early childhood with respect to maternal factors including cognition, childhood growth patterns, infections and socioeconomic factors.
- The child development/cognition measure was administered by a single psychologist at each time point.
- Limitation includes a comparatively small sample size and urban slum setting in South India of the birth cohort, which has to be kept in mind while generalising.

## Introduction

 The first 1000 days of human neurodevelopment are critical, as early childhood development is predictively associated with childhood cognition and overall human potential.^1 2^ These first 1000 days are characterised by neuronal proliferation, migration, differentiation, growth, synapsis and myelination and guide the maximum brain growth in humans. It is critical to monitor for and evaluate any setbacks to child development at this early child development stage, so that early appropriate support can be provided. Child development assessment measures such as Bayley Scales of Infant and Toddler Development (BSID) and Griffiths Mental Developmental Scales (GMDS) are used in early childhood and can be used predictively for future cognitive ability, which can guide overall human potential.^3^,^5^ These tools measure child development domains such as gross motor development, fine motor development, expressive and receptive language and communication skills, early cognition, personal-social skills and practical reasoning. Though, child developmental milestones follow a predictable pattern, cultural factors, parenting practices and local home and community milieu can alter the age of attainment of these milestones.^5 6^ Moreover, developmental assessment measures such as BSID and GMDS are developed in high-income countries, and information about the stability and predictability of these measures in low- and middle-income (LMIC) countries is limited.^7^ Understanding the stability and predictability of early childhood development measures in different populations is important not just for cultural validation and psychometric property understanding but also for comprehending the significance of early childhood findings for later childhood and adult functioning in diverse settings. Potential differences can emerge in disparate or divergent situations from where a measure was originally intended for.

Studies from high-income cohorts have shown varying results in the stability of BSID in early childhood.^8 9^ In LMIC settings, an earlier evaluation had shown that developmental assessments in the first year of life had poor stability in Indonesia with some improvement in stability and predictability after 18 months of age.^10^ In a recent article, BSID-III showed poor stability in early childhood in repeated measures in a prospective cohort analysis in Nepal.^7^

Predictability analysis of childhood cognition from early childhood developmental measures has given mixed results. Predictive validity is the competency of a psychometric measure or tool to predict an individual performance in the future. A meta-analysis showed BSID mental development scores strongly predicted later childhood cognition in very low birth weight/very preterm babies with an explained variance of 37%, while motor scores predicted 12% variance for later motor ability.^11^ For typically-developing babies born full-term, a follow-up study in Sweden concluded that BSID scores at 2.5 years of age were an insufficient predictor of childhood cognition at 6.5 years.^12^ Analysing LMIC studies, while the Indonesian study showed poor cognition predictability of BSID assessment before 18 months of age, a language test development study in Bangladesh showed BSID scores around 1 year of age had a correlation of 0.37 with cognition at 5 years of age.^10 13^ The recent Nepal prospective cohort analysis displayed poor predictability of BSID with cognition analysis at 4 years of age.^7^

Given the criticality of the first 1000 days, the relevance of appropriate developmental assessments in this early childhood stage and the paucity of their stability and predictability analyses especially in LMIC settings, the objective of the present study was to analyse stability and predictive validity of BSID using an existing birth cohort follow-up study in Vellore, South India. It is postulated that BSID will show a moderate correlation for both stability in early childhood and predictability with later childhood cognition at 5, 7 and 9 years of age.

## Methods

### Study design and participants

The current analysis is a substudy of the Indian birth cohort followed for the Aetiology, Risk Factors and Interactions of Enteric Infections and Malnutrition and the Consequences for Child Health and Development (MAL-ED) Network, a prospective longitudinal cohort study conducted in eight countries across the world.^14^ The Indian study site included urban slum dwelling areas, located in Salavanpet, Old Town, and nearby areas in Vellore, South India.^15^ From the available population of 12 000 surveyed in the study area, all pregnant women were identified and invited to join the MAL-ED study. The exclusion criteria were another child already enrolled in the MAL-ED study, plans to migrate during the original study duration (birth until 2 years of age), multiple pregnancies, newborn with medical conditions and absence of informed consent. Further childhood risks including meningitis and head trauma were not excluded. Among the follow-up cohort, there was no incidence of any neurodevelopmental disorder including autism. Babies of mothers who consented were enrolled at an average (range) age of 10.08 (0–16) days. The original birth cohort recruitment between March 2010 and February 2012 enrolled 251 children; whose baseline demographic data was akin to that of the study population.^15^ In addition to semi-weekly morbidity surveillance in early childhood, development assessment was done for the cohort at 6, 15, 24 and 36 months of age. The same birth cohort was further followed-up at 5, 7 and 9 years of age for growth and cognitive assessments. Details of this birth cohort follow-up are already published elsewhere.^16^,^18^ This MAL-ED birth cohort has been used for the current analysis as this cohort was recruited from a single geographical community region and the loss to follow-up did not change the cohort demographically. Follow-up studies were conducted in the same geographical areas and included all children available at that time point. There was no difference in recruitment or follow-up plan.

Informed consent for each stage, approved by the institutional review board had an information sheet about the study, and participants were free to choose all or some components of the study—for example, parent could decide about either one or all 9-year assessments namely growth assessment, child cognition assessments, blood tests and MRI brain neuroimaging. The parent gave written signed consent in the presence of the study psychologist who took the consent in the presence of a witness, who also signed the consent form. Additional verbal assent was obtained from the child before each test.

### Patient/public involvement

The urban slum areas from where the current cohort is recruited are one of the urban areas supported by Christian Medical College Vellore through its Low-Cost Effective Care Centre. All studies in this area are discussed with community leaders and their inputs incorporated into the plans for effective conduct. Community leaders agreed with researchers on the outcome measurement of growth and cognition of children. Field workers/supervisors of this study were recruited from the local community. Individual assessments and reports were provided to families, and those needing medical attention supported with concessional/free treatment in this community. We conduct periodic outreach child health clinics and information discussion meetings in this area for our study families/children and extended community with their inputs incorporated into future conduct of studies.

### Measures

### The Bayley Scales of Infant and Toddler Development-III

The BSID-III assesses child development between 1 and 42 months of age in the following domains: gross motor, fine motor, receptive language, expressive language and cognition.^3^ The BSID scale was adapted, translated and piloted before the study and the measure used for the current analysis was validated in Bangladesh.^19 20^ For each age group setting, only a single trained psychologist did the assessments with a window period of ±2 weeks. A clearly demarcated area in the community clinic was modified child-friendly with minimum distractions and all assessments were conducted in this arena. With additional parental permission, 5% of all assessments including BSID-III were video-recorded and reviewed by the supervising psychologist locally with further review by the central psychologist team for quality control purposes.^19^ All BSID-III forms were double-checked for errors including administrative, coding and calculation errors.

### The Wechsler Preschool Primary Scales of Intelligence – third edition

We used the Wechsler Preschool Primary Scales of Intelligence – third edition (WPPSI-III) to assess cognition at the preschool age of 5 years of age. After appropriate cultural adaptation, translation and piloting, one community psychologist administered this measure in the community clinic.^21^ The cognitive domains included verbal, performance and processing speed and raw scores yielded domain scores and further Full-Scale Intelligence Quotient (FSIQ).

### The Malin’s Intelligence Scale for Indian Children

The Malin’s Intelligence Scale for Indian Children (MISIC)^22^ adapted from the Wechsler Intelligence Scale for Children (WISC) can be administered to children aged 6–16 years. This scale has verbal, performance and full-scale intelligence domains similar to WISC and adaptations include but not limited to culturally appropriate pictures and settings. The testing was done in the community clinic in similar settings as described for BSID-III by a single trained psychologist. For the current cohort, we planned MISIC at 7 and 9 years of age with a window period of +2 months. As per the procedure manual, verbal and performance raw scores were converted to corresponding quotients—performance intelligence quotient and verbal intelligence quotient, respectively, and combined scales FSIQ.

### Public involvement statement

The urban slum areas from where the current cohort is recruited are one of the urban areas supported by CMC through its Low-Cost Effective Care Centre. All studies in this area are discussed with community leaders and their inputs incorporated into the plans for effective conduct. Community leaders agreed with researchers on the outcome measurement of growth and cognition of children. Field workers/supervisors of this study were recruited from the local community. Individual assessments and reports were provided to families, and those needing medical attention supported with concessional/free treatment in this community. We conduct periodic outreach child health clinics and information discussion meetings in this area for our study families/children and extended community with their inputs incorporated into future conduct of studies.

### Statistical analysis

Data management for the original MAL-ED study was done by the Data Coordinating Center established at the Fogarty International Center.^14^ Categorical variables such as socio-demographic characteristics of the cohort were summarised using percentages. Continuous variables such as domain scores of BSID and MISIC scales were presented as mean (SD), after checking for normality. Pearson statistics was used to assess the correlation between domain scores of BSID across time points of measurements as well as with cognition measurements at 5, 7 and 9 years of age. Correlations were expressed as r values. Intraclass correlation coefficient (ICC) and concordance correlation coefficient (CCC) measures were used to express agreement between domain scores of BSID across different time points of assessment (6 vs 36 months, 15 vs 36 months and 24 vs 36 months).

Linear regression analysis was performed to measure the magnitude of change in domain scores of cognition scale at 5, 7 and 9 years, with respect to child development scores measured at 6, 15, 24 and 36 months of age. Multivariable regression analyses were computed having cognition domain scores at 5, 7 and 9 years as separate outcomes. Model fitness was assessed using adjusted R^2^ statistics and p value<0.05 was considered as statistically significant. Stata V.14 StataCorp 2015 Stata Statistical Software: Release 14 (StataCorp College Station, Texas, USA) was used to perform statistical analysis.

## Results

The MAL-ED birth cohort at the Vellore site recruited 251 newborns following a screening of 301 pregnant women from the study area. About 45% of the cohort were males at the time of recruitment. The socio-demographic characteristics of the cohort are summarised in online supplemental table 1 (online supplemental figure 1). No significant difference was observed in terms of sex distribution and socioeconomic status in the cohort between recruitment and follow-up visits, until 9 years of age.^17^ In the cohort, 216 (86.06%) children had complete data points for BSID scores at 6, 15, 24 and 36 months of age. Further, 212 (84.46%), 189 (75.30%) and 203 (80.88%) children had information on cognition measures at 5, 7 and 9 years, respectively. Mean (SD) IQ scores of cognition, language, motor and social domains of BSID measurement at 6 months were 102.43 (9.93), 98 (8.38), 106.04 (14.08) and 115.69 (13.4), respectively. On comparing this with subsequent BSID measurements at 15, 24 and 36 months of age, a significant decline was observed in mean scores for all four domains of child development.^18^ Mean (SD) IQ scores of verbal, performance, processing speed and full-scale domains of the WPPSI scale were 80.95 (6.46), 84.64 (9.06), 101.27 (16.84) and 83.56 (8.58), respectively. Mean (SD) IQ scores under verbal, performance and total IQ domains of the MISIC scale were 95.54 (11.13), 82.05 (16.97) and 88.88 (12.18) at 7 years and, 93.98 (9.72), 91.45 (13.06) and 92.71 (10.28), respectively at 9 years.

Overall, ICC values of the domains of the BSID scales suggested a weak correlation existing between measurements made at 6, 15, 24 and 36 months of age. Comparatively, ICC values for language (0.21 (95% CI: 0.14 to 0.28)) and motor (0.24 (95% CI: 0.17 to 0.31)) domains were more stable than cognition and social domains of the BSID scales (table 1). A similar pattern was observed when the CCC measure was used to assess the agreement between BSID domain scores measured at infancy and early toddlerhood (15 and 24 months) and during later toddler ages (36 months). Motor and language domains had higher CCC values of 0.26 (95% CI: 0.15 to 0.37) and 0.28 (95% CI: 0.20 to 0.36), respectively, when concordance was assessed for BSID scores between 24 and 36 months. Higher CCC values were noted, while agreement was computed between domain scores at closer time points (24 and 36 months), than those farther apart (6 and 36 months) (table 2).

**Table 1:** Intraclass correlation of domain scores of Bayley Scales across time points of measurement in children of MAL-ED cohort (n=216)

Domains of Bayley Scales	Intraclass correlation coefficient (95% CI)
Cognition	−0.05 (−0.10 to 0.01)
Language	**0.21(0.14to0.28)**
Motor	**0.24(0.17to0.31)**
Social	−0.10 (−0.13 to −0.05)

Values in bold fonts represent statistical significance.

MAL-EDAetiology, Risk Factors and Interactions of Enteric Infections and Malnutrition and the Consequences for Child Health and Development

**Table 2:** Concordance correlation coefficient (CCC) of domain scores of Bayley Scales between the time points of measurement in children of MAL-ED cohort (n=216)

Time points	Parameters	Domains of Bayley Scales			
		Cognition	Language	Motor	Social
6 vs 36 months	CCC (95% CI)	**0.05(0.02to0.08)**	0.06 (–0.03 to 0.15)	**0.18(0.09to0.27)**	**−0.06(–0.09to–0.03)**
	Difference average	15.90	4.88	6.85	17.04
	95% limits of agreement	−3.72 to 35.53	−12.98 to 22.73	−20.66 to 34.36	−12.43 to 46.51
15 vs 36 months	CCC (95% CI)	0.01 (–0.03 to 0.04)	**0.12(0.03to0.20)**	**0.25(0.13to0.38)**	**0.06(0.04to0.09)**
	Difference average	14.95	4.97	0.92	18.08
	95% limits of agreement	−5.62 to 35.53	−13.01 to 22.95	−17.91 to 19.76	−3.57 to 39.73
24 vs 36 months	CCC (95% CI)	**0.09(0.01to0.17)**	**0.28(0.20to0.36)**	**0.26(0.15to0.37)**	**0.11(0.08to0.15)**
	Difference average	6.00	4.917	4.07	15
	95% limits of agreement	−9.02 to 21.01	−11.01 to 20.84	−14.50 to 22.64	−2.25 to 32.25

Values in bold fonts represent statistical significance.

MAL-EDAetiology, Risk Factors and Interactions of Enteric Infections and Malnutrition and the Consequences for Child Health and Development

The correlation matrix between domains of BSID at 6, 15, 24 and 36 months and cognition measurements at 5, 7 and 9 years is shown in online supplemental table 2. Poor correlation was observed while BSID scores were compared within its domains at specific time points as well as between the time points of assessment (6–36 months), with correlation coefficients (r) ranging between −0.06 (between 6 months language and 24 months cognition) and 0.59 (between 15 months language and 15 months cognition). Further assessment of the correlation between BSID measurements over 6–36 months and 5-year cognition showed that strength of correlation was best (moderate positive correlation) for BSID scores at 36 months (r: 0.40–0.49) and the weakest correlation (low) was seen with BSID scores at 6 months (r: 0.21–0.30). Correlation between later childhood cognition at 5, 7 and 9 years showed a good correlation with r value ranging between 0.70 and 0.79 (online supplemental table 2).

Regression analysis between 5-year cognition and child development scores showed that BSID scores at 36 months predicted FSIQ at 5 years better (R^2^ - 28.63%), than BSID measurements at 6, 15 and 24 months with R^2^ values of 8.43%, 11.92% and 25.86%, respectively. The language domain of the BSID scale was a significant predictor for FSIQ at 5 years with beta coefficients ranging between 0.17 and 0.33 between 15 and 36 months of age. Further, BSID scores at 24 months were found to predict the performance domain (R^2^ - 26.74%) of 5 years cognition better than verbal (R^2^ - 15.74%) or processing speed domain (R^2^ - 13.23%) (table 3).

**Table 3:** Relationship between childhood development scores and cognition attainment at 5 years in children of MAL-ED cohort (n=208)

Domain	Cognition at 5 years, β coefficients (95% CI)			
	Verbal IQ	Performance IQ	Processing speed IQ	Full-scale IQ
6 months				
Cognition	0.05 (–0.08 to 0.17)	−0.06 (–0.23 to 0.11)	−0.03 (–0.33 to 0.27)	0 (–0.16 to 0.16)
Language	0 (–0.13 to 0.13)	0.08 (–0.10 to 0.25)	**0.46(0.15to0.77)**	0.10 (–0.06 to 0.27)
Motor	0.06 (–0.03 to 0.15)	**0.20(0.08to0.32)**	**0.36(0.15to0.57)**	**0.17(0.06to0.28)**
Social	0.01 (–0.07 to 0.09)	**−0.11(–0.21to–0.01)**	−0.10 (0.28 to 0.08)	−0.06 (–0.15 to 0.04)
R^2^ value	2.2%	6.75%	17.08%	8.43%
15 months				
Cognition	−0.01 (–0.13 to 0.11)	0.02 (–0.15 to 0.19)	**0.32(0.01to0.62)**	0.06 (–0.09 to 0.22)
Language	**0.22(0.09to0.35)**	0.09 (–0.09 to 0.27)	0.14 (–0.18 to 0.47)	**0.17(0to0.33)**
Motor	0.05 (–0.08 to 0.18)	**0.22(0.05to0.40)**	**0.40(0.07to0.72)**	0.19 (0.02 to 0.35)
Social	−0.01 (–0.09 to 0.07)	0.03 (–0.07 to 0.14)	−0.03 (–0.22 to 0.17)	0.01 (–0.09 to 0.11)
R^2^ value	9.18%	7.07%	13.64%	11.92%
24 months				
Cognition	0.10 (–0.04 to 0.24)	0.21 (0.04 to 0.39)	−0.11 (–0.48 to 0.25)	0.16 (–0.01 to 0.34)
Language	**0.24(0.13to0.34)**	**0.31(0.18to0.45)**	**0.56(0.29to0.83)**	**0.33(0.20to0.46)**
Motor	0.01 (–0.09 to 0.11)	**0.09(–0.04to0.22)**	**0.39(0.12to0.65)**	0.12 (0 to 0.25)
Social	0.04 (–0.05 to 0.13)	0.13 (0.02 to 0.25)	−0.03 (–0.27 to 0.20)	0.08 (–0.03 to 0.19)
R^2^ value	15.74%	26.74%	13.23%	25.86%
36 months				
Cognition	0.15 (–0.07 to 0.36)	0.20 (–0.09 to 0.50)	**0.81(0.25to1.38)**	0.31 (0.04 to 0.58)
Language	**0.44(0.21to0.67)**	**0.43(0.12to0.74)**	**0.94(0.35to1.53)**	**0.58(0.30to0.86)**
Motor	0.13 (0 to 0.26)	**0.28(0.11to0.46)**	0.26 (–0.07 to 0.59)	**0.23(0.07to0.39)**
Social	0.02 (–0.20 to 0.23)	0.20 (–0.08 to 0.49)	−0.40 (–0.95 to 0.14)	0.07 (–0.19 to 0.33)
R^2^ value	18.86%	21.79%	17.77%	28.63%

Values in bold fonts represent statistical significance.

IQintelligence quotientMAL-EDAetiology, Risk Factors and Interactions of Enteric Infections and Malnutrition and the Consequences for Child Health and Development

Similar to the 5-year analysis, BSID scores at 36 months predicted FSIQ at 7 and 9 years better with R^2^ values of 26.01% and 23.32%, compared with other time points. Language and motor domains of BSID were found to be significant predictors of FSIQ at 7 years with beta coefficients ranging between 0.27–0.83 and 0.20–0.37, respectively. However, this association was weakened in the 9-year analysis, where beta coefficients of language and motor domains ranged between 0.16–0.51 and 0.14–0.30, respectively (table 4).

**Table 4:** Relationship between childhood development scores and cognition attainment at 7 and 9 years in children of MAL-ED cohort

Domain	Cognition at 7 years (n=189), β coefficients (95% CI)			Cognition at 9 years (n=200), β coefficients (95% CI)		
	Verbal IQ	Performance IQ	Full-scale IQ	Verbal IQ	Performance IQ	Full-scale IQ
6 months						
Cognition	−0.05 (–0.29 to 0.19)	−0.32 (–0.68 to 0.04)	−0.18 (–0.44 to 0.07)	−0.06 (–0.26 to 0.13)	−0.21 (–0.47 to 0.04)	−0.14 (–0.34 to 0.07)
Language	0.19 (–0.04 to 0.42)	**0.43(0.07to0.78)**	**0.32(0.06to0.57)**	0.11 (–0.09 to 0.31)	0.20 (–0.06 to 0.46)	0.16 (–0.05 to 0.36)
Motor	0.13 (–0.03 to 0.30)	**0.26(0.01to0.51)**	**0.20(0.02to0.38)**	0.10 (–0.03 to 0.24)	**0.31(0.13to0.49)**	**0.21(0.07to 0.35)**
Social	−0.10 (–0.24 to 0.04)	−0.05 (–0.26 to 0.16)	−0.08 (–0.23 to 0.07)	−0.03 (–0.15 to 0.08)	−0.13 (–0.29 to 0.02)	−0.08 (–0.20 to 0.04)
R^2^ value	2.64%	5.04%	5.97%	0.8%	7.49%	5.47%
15 months						
Cognition	−0.06 (–0.28 to 0.17)	0.20 (–0.14 to 0.54)	0.07 (–0.17 to 0.31)	−0.02 (–0.22 to 0.17)	0.18 (–0.07 to 0.43)	0.08 (–0.12 to 0.27)
Language	**0.26(0.02to0.50)**	0.24 (–0.12 to 0.60)	**0.27(0.01to0.52)**	**0.21(0to0.41)**	0.17 (–0.10 to 0.44)	0.19 (–0.02 to 0.40)
Motor	0.13 (–0.11 to 0.36)	0.30 (–0.05 to 0.65)	0.22 (–0.03 to 0.47)	0.09 (–0.11 to 0.30)	0.22 (–0.04 to 0.49)	0.16 (–0.05 to 0.37)
Social	0 (–0.14 to 0.13)	0.08 (–0.13 to 0.28)	0.04 (–0.10 to 0.19)	0.72 (–0.05 to 0.19)	0.04 (–0.11 to 0.19)	0.06 (–0.07 to 0.18)
R^2^ value	3.85%	9.86%	10.95%	4.95%	9.59%	9%
24 months						
Cognition	0.01 (–0.23 to 0.25)	0.18 (–0.18 to 0.55)	0.11 (–0.15 to 0.36)	0.16 (–0.06 to 0.38)	**0.30(0.01to0.59)**	**0.23(0to0.46)**
Language	**0.50(0.31to0.69)**	**0.40(0.11to0.69)**	**0.45(0.25to0.66)**	**0.31(0.15to0.48)**	**0.36(0.14to0.57)**	**0.34(0.17to0.50)**
Motor	−0.05 (–0.23 to 0.13)	**0.57(0.30to0.84)**	**0.28(0.09to0.47)**	0.05 (–0.11 to 0.21)	**0.23(0.03to0.44)**	0.14 (–0.02 to 0.30)
Social	0.03 (–0.13 to 0.19)	−0.10 (–0.34 to 0.14)	−0.02 (–0.19 to 0.15)	−0.05 (–0.19 to 0.09)	−0.08 (–0.27 to 0.11)	−0.06 (–0.21 to 0.08)
R^2^ value	14.66%	17.08%	19.3%	10.83%	14.66%	16.04%
36 months						
Cognition	**0.42(0.03to0.80)**	0.19 (–0.42 to 0.81)	0.25 (–0.17 to 0.66)	0.24 (–0.10 to 0.57)	0.38 (–0.07 to 0.83)	0.31 (–0.04 to 0.65)
Language	**0.84(0.45to1.22)**	**0.78(0.16to1.40)**	**0.83(0.41to1.25)**	**0.60(0.26to0.95)**	0.42 (–0.05 to 0.88)	**0.51(0.15to0.87)**
Motor	0.07 (–0.16 to 0.29)	**0.66(0.29to1.02)**	**0.37(0.13to0.62)**	0.12 (–0.09 to 0.32)	**0.49(0.22to0.76)**	**0.30(0.09to0.51)**
Social	0.16 (–0.21 to 0.52)	−0.04 (–0.62 to 0.55)	0.10 (–0.30 to 0.49)	0.30 (–0.03 to 0.62)	0.25 (–0.19 to 0.68)	0.27 (–0.06 to 0.60)
R^2^ value	24.21%	17.66%	26.01%	19.3%	19.47%	23.32%

Bold values indicate statistical significance.

Outcome 1: Ddomain scores of cognition measure at 7 years and outcome 2: Ddomain scores of cognition measure at 9 years.

IQintelligence quotientMAL-EDAetiology, Risk Factors and Interactions of Enteric Infections and Malnutrition and the Consequences for Child Health and Development

## Discussion

This birth cohort follow-up study from Vellore, South India, had BSID assessments at 6, 15, 24 and 36 months and cognitive assessments at 5, 7 and 9 years of age. Follow-up cohorts at each time point were comparable to the original birth cohort in terms of demographic characteristics. Analysis showed low stability for BSID across time points with the best ICC and CCC scores shown for language and motor domains, and between 24 and 36 months of age. Overall, early BSID scores showed poor predictability of preschool and school-age cognition with BSID scores at 36 months showing the best correlation with later cognition. The current analysis adds to existing evidence not just in the poor stability of BSID scores, but also in the poor predictability of early BSID scores with preschool cognition at 5 years and school-age cognition at 7 and 9 years of age. BSID scores at 36 months predicted FSIQ better at 5, 7 and 9 years with R^2^ ranging from 23.32% to 28.63%. This is one of the few studies in an LMIC setting that provides long-term implications of repeated measures of BSID scores on cognition during childhood.

Results from this birth cohort study are similar to those reported from Nepal, Pakistan and Bangladesh.^7 13 23^ The current study showed better stability for language and motor domains for repeated BSID measurements from infancy to preschool years, with ICC of 0.21 and 0.24, similar to the Nepal study that reported ICC of 0.19 and 0.22, respectively.^7^ With reference to concordance between BSID measures across time points, this study observed better concordance for measurements between closer time points of 24 and 36 months, with respect to motor and language domains, reporting CCC of 0.26 and 0.28, in line with the Nepal study having CCC of 0.20 and 0.36, respectively.^7^ Predictability of IQ at 5 and 7 years based on BSID measure at 24 months was better in the current study done from urban India with a variance of 26% and 20%, compared with a rural cohort in Pakistan with a lesser variance of 15% and 1%, respectively.^23^ Having disadvantaged children from rural setting, with a higher rate of stunting of 60% was reported as a plausible reason for the lesser predictability of IQ in later childhood based on BSID at 24 months in the Pakistan cohort.^23^

The BSID being the most commonly used child development measure in both child research and clinical practices has faced criticism with regard to overestimation of child development,^24^ and in turn underestimating developmental delay and disability in both low and high-risk populations including preterm infants.^8 9 25 26^ Low stability and poor predictability of BSID in early childhood with future cognitive measures as shown in the current analysis was reported from both high and low resource settings.^7^,^926^ One reason could be that development assessments in early childhood concentrates on the achievement of specific development milestones constructed based up on systemic observations in typically developing infants mainly in high-income countries. Infants in LMIC settings face socioeconomic disadvantage and malnutrition, affecting the timing of achievement of development milestones. Thus, developing a context-specific tool incorporating infant development components of habituation, novelty preference and reaction time in development assessment can improve the stability of these tools and contribute to better predictability with future intelligence in these children.^12^

Developmental assessments in very young children less than 3 years of age can be challenging in terms of appropriate measures, assessment settings, engaging very young physically active children, sustaining attention for relevant activities and cultural adaptability in new settings. Developmental measures use domains such as motor, language and social skills and cognition, while cognitive measurements use verbal and performance domains along with processing speed.^3 4 22 27^ Early childhood development and later cognitive development are evolving and dynamic with multiple inter-related influential factors such as childhood experiences, socioeconomic, home and learning environments, maternal/parental interactions, infections and nutritional and micronutrient deficiencies. This evolution of constructs of child development and cognition in children can also cause poor stability and predictability of developmental measures such as BSID as seen in this study.

The early childhood development in this cohort showed a significant declining trend, which was explored in detail in another analysis highlighting stunting, socioeconomic status (SES), home environment and blood iron status as substantial predictors.^18^ The similar declining trend in developmental scores in language, fine motor and visual perceptual skills were found in another rural Indian study evaluating early child development^28^ and in cognitive, language and fine motor scales in preterm infants in an academic setting in the USA.^26^ Persistent exposure to risks such as macro-nutritional and micro-nutritional deficiencies, environmental toxins and suboptimal socioeconomic and home environments might cause an overall deterioration in developmental potential. It must be noted that this LMIC cohort had around 45% children stunted at 24 months of age,^17^ more than 40% iron deficient at 15 and 24 months^29^ and 95% and 97%, respectively, had high blood lead levels at 24 and 36 months.^29^ It should be highlighted that the BSID-III used in the current analysis was sensitive to capture these changes in early childhood and the updated BSID-IV also addresses the overestimation of child development.

The BSID scores at 3 years had better predictability of 5-year, 7-year and 9-year cognition. As 2 years of age is the culmination of the first 1000 days of life, developmental assessments at this age are given more importance. In the current analysis, 3-year BSID scores had better predictability of future cognition scores than 2-year values. The BSID scores at 2.5 years were predictive of WISC FSIQ (24% variance) at 6.5 years in a follow-up study in Sweden,^12^ while in a Pakistani study, BSID scores at 2 years predicted a 15% variance of WPPSI scores at 4 years and variance of 1–16% for WISC domain scores at 8 years of age.^23^ Language and motor domains of BSID measurements at 3 years had a significant association with FSIQ measurements at 5, 7 and 9 years in our study. Similar findings were noted in a study from Bangladesh with a significant correlation between BSID language scores at 1 year and cognitive ability at 5 years.^13^ Language skills can act as a medium through which a child interacts and learns both at home and school driving academic achievements.

There are limitations to the present study. We had a comparatively small sample size, and the cohort was from an urban slum setting—this should be kept in mind while generalising current study findings. Though BSID-III and MISIC were validated for LMIC settings, WPPSI is not a validated measure. Strengths of this longitudinal follow-up study include standardised assessments done at each time point by a single trained psychologist to prevent inter-rater reliability variations, good local and global quality checks for assessments and good data granularity in early childhood including growth, infections, SES and maternal factors.

Future implications of the present study would be to consider the use of developmental measures in sequential settings rather than at a single point while evaluating developmental level in early childhood. From a public health perspective, the window for ideal child development assessment appears 24–36 months of age. Appropriate child development screening and assessment tools can act as enablers in community, academic and referral child health settings, where they along with other relevant clinical assessments can highlight risks ensuring appropriate early intervention.^8^

## Conclusions

Child development assessments such as BSID are important not just to compare child development against a standard, but also to evaluate outcomes in early childhood research settings, especially in randomised controlled intervention trials. As seen in this study, the poor stability of BSID in early childhood warrant caution in the predictive projection of early childhood assessments. Better predictability of future cognition of 36 months’ BSID scores highlights its advantage over 24 months. Generalisations, overall interpretations and predictability proposals, including cognition and human potential from early childhood development assessments need to be tempered in the background of the current analysis and other literature evidence.

## supplementary material

10.1136/bmjopen-2023-082624 online supplemental figure 1

10.1136/bmjopen-2023-082624 online supplemental table 1

## Data Availability

Data are available in a public, open access repository. Data are available upon reasonable request.
