# Supplementary material for: Stability and predictability of Bayley Scales of Infant and Toddler Development: evidence from a south Indian birth cohort prospective study
Source: BMJ Open. 2024 Nov 19;14(11):e082624. doi: 10.1136/bmjopen-2023-082624 (PMC11580237; doi:10.1136/bmjopen-2023-082624)
Supplement: online supplemental figure 1 [file bmjopen-14-11-s001.pdf]

Supplementary Figure 1: Study profile

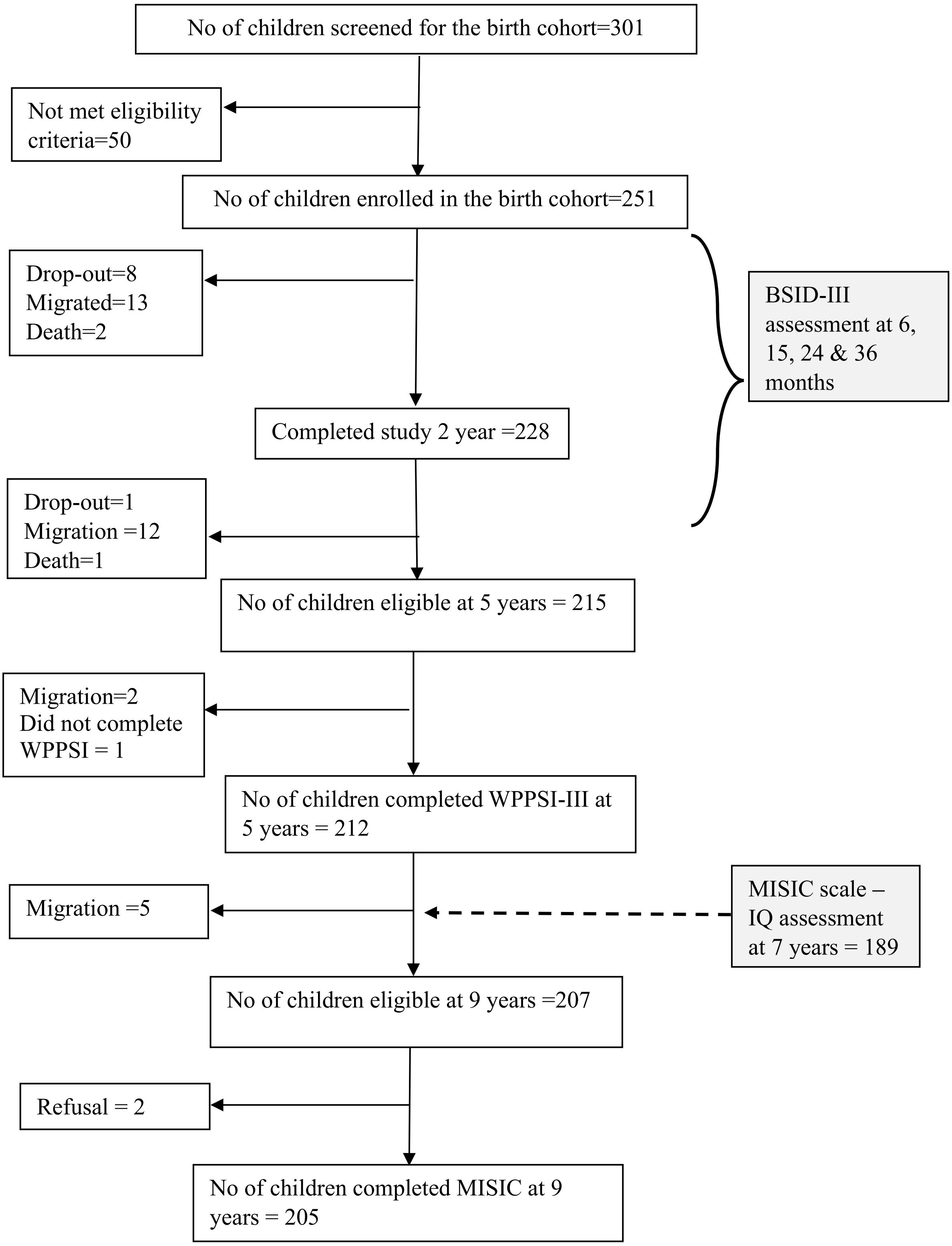

BSID: The Bayley Scales of Infant and Toddler Development-III; WPPSI: The Wechsler Preschool Primary Scales of Intelligence; and MISIC: The Malin's Intelligence Scale for Indian Children
