# Supplementary material for: Stability and predictability of Bayley Scales of Infant and Toddler Development: evidence from a south Indian birth cohort prospective study
Source: BMJ Open. 2024 Nov 19;14(11):e082624. doi: 10.1136/bmjopen-2023-082624 (PMC11580237; doi:10.1136/bmjopen-2023-082624)
Supplement: online supplemental table 1 [file bmjopen-14-11-s002.pdf]

Supplementary Table 1: Characteristics of children of MAL-ED cohort at recruitment and 2 years

**Baseline**	Gender	**N**	**n (%)**
	Male	251	113 (45.0)
	Female		138 (55.0)
	Children who had birth weight < 2.5 kg		43 (17.13)
	Children with height-for-age Z scores (< -2SD)		41 (16.34)
	Age of the mother[#]	249	
	< 23 years		103 (41.37)
	≥ 23 years		146 (58.63)
	Socioeconomic status	235	
	Low (WAMI <33rd percentile)		71 (30.2)
	High (WAMI ≥33rd percentile)		164 (69.8)
	Maternal cognition raw scores*, mean (SD)		43.90 (10.49)
**At 2 years**	Children with anaemia (Hb < 11 gm/dl)	225	96 (42.67)
	Children with height-for-age Z scores (< -2SD)	227	101 (44.30)
	Children with weight-for-age Z scores (< -2SD)		81 (35.53)

MAL-ED - Etiology, Risk Factors and Interactions of Enteric Infections and Malnutrition and the Consequences for Child Health and Development; WAMI - Water and sanitation, Assets, Maternal education and household Income

Supplementary Table 2: Correlation between Bayley scale scores of early childhood, WPPSI scale at 5 years and MISIC scale measurements at 7 and 9 years in children of MAL-ED cohort (n=195)

		**6 months**			**15 months**			**24 months**			**36 months**			**5 y**	**7 y**	**9 y**
		Cognition	Language	Motor	Cognition	Language	Motor	Cognition	Language	Motor	Cognition	Language	Motor	FSIQ	FSIQ	FSIQ
**6 mo**	Cognition	1														
	Language	**0.51**	1													
	Motor	**0.69**	**0.53**	1												
**15 mo**	Cognition	**0.28**	**0.36**	**0.36**	1											
	Language	**0.26**	**0.43**	**0.31**	**0.59**	1										
	Motor	**0.23**	**0.32**	**0.39**	**0.57**	**0.53**	1									
**24 mo**	Cognition	0.05	-0.06	0.13	0.13	0.04	**0.14**	1								
	Language	0.13	0.07	**0.17**	**0.31**	**0.34**	**0.26**	**0.43**	1							
	Motor	0.13	**0.16**	**0.27**	**0.38**	**0.24**	**0.35**	**0.31**	**0.28**	1						
**36 mo**	Cognition	**0.21**	**0.14**	**0.21**	0.02	0.12	0.08	**0.15**	**0.23**	**0.15**	1					
	Language	0.11	0.10	**0.17**	**0.19**	**0.19**	**0.20**	**0.30**	**0.43**	0.27	**0.49**	1				
	Motor	**0.15**	0.11	**0.26**	**0.22**	**0.18**	**0.26**	**0.23**	**0.24**	0.30	**0.42**	**0.46**	1			
**5 y**	FSIQ	**0.21**	**0.22**	**0.30**	**0.28**	**0.32**	**0.32**	**0.36**	**0.47**	**0.28**	**0.40**	**0.49**	**0.41**	1		
**7 y**	FSIQ	0.08	**0.22**	**0.21**	**0.27**	**0.32**	**0.30**	**0.26**	**0.41**	**0.32**	**0.35**	**0.48**	**0.41**	**0.70**	1	
**9 y**	FSIQ	0.09	**0.17**	**0.23**	**0.25**	**0.30**	**0.27**	**0.30**	**0.40**	**0.25**	**0.36**	**0.42**	**0.38**	**0.70**	**0.79**	1

Values in bold fonts represent statistical significance.
