# Supplementary material for: Stability and predictability of Bayley Scales of Infant and Toddler Development: evidence from a south Indian birth cohort prospective study
Source: BMJ Open. 2024 Nov 19;14(11):e082624. doi: 10.1136/bmjopen-2023-082624 (PMC11580237; doi:10.1136/bmjopen-2023-082624)
Supplement: Supplementary file 3 [file bmjopen-2023-082624.reviewer_comments.pdf]

PEER REVIEW HISTORY

BMJ Open publishes all reviews undertaken for accepted manuscripts. Reviewers are asked to complete a checklist review form and are provided with free text boxes to elaborate on their assessment. These free text comments are reproduced below.

ARTICLE DETAILS

Title (Provisional)

Stability and predictability of Bayley Scales of Infant Development: Evidence from a south Indian birth cohort prospective study

Authors

Koshy, Beena; Srinivasan, Manikandan; Scharf, Rebecca; Strand, Tor A; Mohan, Venkata Raghava; Beulah, Rachel; John, S; Muliyil, Jayaprakash; Kang, Gagandeep

---

VERSION 1 - REVIEW

**Reviewer**	**1**
**Name**	**Schonhaut, Luisa**
**Affiliation**	**Clinica Alemana de Santiago SA**
**Date**	**24-Dec-2023**
**COI**	**I have no conflict of interest**

It is an interesting study, well written and well referenced. Just a few suggestions.

1. The objectives could be more explicitly stated in the abstract and at the end of the introduction of the paper.

2. Children were assessed with BSID at 6-15-24 and 36 months, with WPSSI at 5 years, and then with MISIC at 7 and 9 years. The stability of the BSID at these points (6-15-24 and 36 months) and its predictive ability in the long term (5,7 and 9 years) are measured, with analyses very similar to Kvestad I, et al. The stability of the Bayley scales in early childhood and its relationship with future intellectual abilities in a low to middle income country. Early Hum Dev. 2022;170:105610.

There is a previous analysis of developmental trends in early childhood in the first 3 years of life using BSID, from the same authors, that shows "Early childhood development in this cohort showed a significant decreasing trend", which certainly affects the long-term predictive ability of the BSID. Koshy B, et al. BMC Public Health. 2021;21(1):1083. I wonder what adds to the knowledge the stability of BSID in this population.

Another way of looking at the data could be the analysis of the cognitive trajectory of this sample of 251 LMIC children (considering that they were assessed at different ages with different tests), it seems to me that in the discussion the authors focused on this aspect, that could be deduced from this data.

3. I understand that the data were analyzed as continuous variables, I do not know if it is justified to add cut-off points for risk/delay for each of the scales. It should also be specified whether these scales were validated in India.

If the tests used were not validated in India, this should be shown as a limitation of the study. I suggest reviewing the way to Kvestad I, et al. 2022 addressed this point.

4. In methodology it should be made explicit if there were specific exclusion criteria for this study (e.g. children with Autism Spectrum Disorder who are non-speakers, I imagine that they cannot be evaluated with WPSSI or MISIC, or children who had serious events that could affect neurodevelopment such as head trauma, meningitis... it should be explained if they considered what to do in those cases (independently if there were no cases)).

Reviewer 2

Name Bhurawala, Habib

Affiliation The University of Sydney Nepean Clinical School, Paediatric Department

Date 24-Jan-2024

COI No competing interests

Thanks for the opportunity to review this paper. The manuscript provides a comprehensive investigation into the stability and predictability of developmental assessments in early childhood in a LMIC setting. The study design and statistical analyses appear robust, and the discussion could be strengthened by providing more detailed comparisons with existing literature. Consider refining some language for clarity, particularly in complex sentences-breaking down to shorter and simpler sentences, and ensure a strong linkage between results and the broader implications for child health research and practice. Few specific areas which needs attention to improve the manuscripts as below. 1. In Introduction- Please expand and clarify, Why stability and predictability are crucial in LMIC settings specifically, acknowledging potential differences compared to high-income settings.2. Briefly clarify the concept of "predictability" in the context of developmental assessments for readers who may not be familiar with the term. 3.Explicitly state how the study's findings may inform public health strategies or interventions in LMICs.4. In Study methods- Please justify the choice of the MAL-ED Network for the birth cohort and explain why this cohort is representative. 5. From Ethical consideration perspective- Please clarify if any modifications

were made to the original cohort recruitment protocol for the follow-up studies.6. Please provide additional details on why the specific urban slum areas in Vellore were chosen, and discuss any potential implications for generalisability. 7 Please specify the process of obtaining informed consent, particularly emphasising how consent was obtained from caregivers and assent from children at 9 years.8.Please provide additional information on the adaptation of MISIC from WISC, ensuring clarity on modifications made for cultural relevance.9 In terms of data management and statistical analysis- Please Specify if there were any specific procedures or considerations during data management, especially since it was done by the Data Coordinating Centre at the Fogarty International Centre.10. Please provide more context for the choice of Pearson correlation. For example, if any assumptions of the Pearson correlation were checked or if there were any concerns regarding the non-normality of continuous variables. 11 Please mention the version of STATA software. 12 Can you pls clarify clarify if there were any significant differences between those who dropped out or didn’t have complete data and those who were included in the analysis.13 Can you please expand and provide more explicit, simple and easy to understand guidance on the clinical implications of your findings. How might these results influence early childhood developmental assessments in clinical practice or research especially in LMIC settings? Thank you.

**Reviewer**	**3**
**Name**	**Varela , Natalia**
**Affiliation**	
**Date**	**21-Feb-2024**
**COI**	**none**

The authors do not adequately justify the necessity of addressing 'stability' in the introduction, and they fall short in providing a solid foundation for employing ICC as an indicator of stability. ICC is commonly utilized to assess inter-rater agreement, reflecting the extent of shared variance among various measures. However, in the context of longitudinal data, the interpretation of common variance can be multifaceted, and the authors do not explicitly articulate the criteria for defining robust common variance. It is crucial to substantiate the use of ICC as a metric for 'stability' in a longitudinal design. Moreover, the introduction should prominently address the rationale behind their interest in stability, as this is of paramount importance. The central concern remains the scientific relevance of documenting stability. If the rationale for this documentation is not clearly articulated, determining an appropriate methodology becomes a challenging task.

Prof. André Plamondon helped to complete the review.

**Reviewer**	**4**
**Name**	**Alexander, Caroline**
**Affiliation**	**Curtin University, School of Occupational Therapy, Sociak Work and Speech Pathology**
**Date**	**18-Jun-2024**
**COI**	**No competing interests to declare**

Thank you for this valuable study exploring the stability of the Bayley Scales and predictability to later cognitive outcomes in childhood in low- middle- income settings. The research question is interesting and worthy of investigation. The manuscript is of high quality, well organised and well written. The clear and transparent description of the study population, study design and methodology and presentation of the results is commended.

I make just a few minor typographical notes and a suggestion for discussion.

1. The authors switch between "BSID" and "Bayley" in text so I suggest reviewing for consistency

2. Page 10 of 30, line 53 – is this meant to read as "more stable"?

3. Page 14 of 30, line- 48 – The BSID scores at 3 years had better predictability of 3- 5- and 7-year cognition – is this meant to read as 5- 7- and 9- year cognition?

4. Finally, the authors note in the discussion there has been concerns raised regarding the overestimation of the child development in the BSID-III. The authors may like to consider noting that the revised Bayley-4 has sought to address this.

VERSION 1 - AUTHOR RESPONSE

Reviewer: 1

1. It is an interesting study, well written and well referenced.

Thank you for your encouraging feedback.

2 The objectives could be more explicitly stated in the abstract and at the end of the introduction of the paper.

Thank you. We have edited in page 2 (abstract) and page 5 correspondingly.

3. Children were assessed with BSID at 6-15-24 and 36 months, with WPSSI at 5 years, and then with MISIC at 7 and 9 years. The stability of the BSID at these points (6-15-24 and 36 months) and its predictive ability in the long term (5,7 and 9 years) are measured, with analyses very similar to Kvestad I, et al. The stability of the Bayley scales in early childhood and its

relationship with future intellectual abilities in a low to middle income country. Early Hum Dev. 2022;170:105610.

There is a previous analysis of developmental trends in early childhood in the first 3 years of life using BSID, from the same authors, that shows "Early childhood development in this cohort showed a significant decreasing trend", which certainly affects the long-term predictive ability of the BSID. Koshy B, et al. BMC Public Health. 2021;21(1):1083. I wonder what adds to the knowledge the stability of BSID in this population.

Thank you. Understanding stability and predictability of early childhood development measures in different populations is important not just for cultural validation and psychometric property understanding but also to comprehend the significance of early childhood findings for later childhood and adult functioning in diverse settings. This comment has been added to introduction – page 4

4. Another way of looking at the data could be the analysis of the cognitive trajectory of this sample of 251 LMIC children (considering that they were assessed at different ages with different tests), it seems to me that in the discussion the authors focused on this aspect, that could be deduced from this data.

Thank you. The focus of this paper is on stability and predictability of BSID

5. I understand that the data were analyzed as continuous variables, I do not know if it is justified to add cut-off points for risk/delay for each of the scales. It should also be specified whether these scales were validated in India.
If the tests used were not validated in India, this should be shown as a limitation of the study. I suggest reviewing the way to Kvestad I, et al. 2022 addressed this point.

Thank you. Unlike Kvestad paper, we did not use any cut-offs for our analysis. The BSID-III we used in India was validated in Bangladesh, which has similar settings as ours. MISIC is Indian adaptation of WISC and thus validated.

6. In methodology it should be made explicit if there were specific exclusion criteria for this study (e.g. children with Autism Spectrum Disorder who are non-speakers, I imagine that they cannot be evaluated with WPSSI or MISIC, or children who had serious events that could affect neurodevelopment such as head trauma, meningitis… it should be explained if they considered what to do in those cases (independently if there were no cases)).
Thank you. This was a birth-cohort follow-up study. Exclusion criteria were provided on page 6 of the manuscript and this cohort excluded multiple pregnancy and newborns with any known medical conditions including perinatal and antenatal risks. Overall, this cohort was a typically developing cohort.

Reviewer: 2

1 Thanks for the opportunity to review this paper. The manuscript provides a comprehensive investigation into the stability and predictability of developmental assessments in early childhood in a LMIC setting. The study design and statistical analyses appear robust, and the discussion could be strengthened by providing more detailed comparisons with existing

literature. Consider refining some language for clarity, particularly in complex sentences-breaking down to shorter and simpler sentences, and ensure a strong linkage between results and the broader implications for child health research and practice. Few specific areas which needs attention to improve the manuscripts as below.

In Introduction- Please expand and clarify, Why stability and predictability are crucial in LMIC settings specifically, acknowledging potential differences compared to high-income settings.

Thank you for your encouraging words. Corresponding changes added – page 4

2. Briefly clarify the concept of "predictability" in the context of developmental assessments for readers who may not be familiar with the term.

Thank you. Corresponding change made – page 5

3.Explicitly state how the study's findings may inform public health strategies or interventions in LMICs.

We believe that this information is implicit in the updated document in that it is preferable to follow children beyond two years when neurocognition is the outcome. This study has highlighted that cognitive development assessments at 24 and 36 months of age are better predictor of later childhood cognition. Therefore, the time period of 24-36 months of age represents crucial age window for public health screening programme to identify children at risk of cognitive delay for providing supportive interventions.

4. In Study methods- Please justify the choice of the MAL-ED Network for the birth cohort and explain why this cohort is representative.

Thank you, Changes made – Page 6

1. From Ethical consideration perspective- Please clarify if any modifications were made to the original cohort recruitment protocol for the follow-up studies.

Follow-up studies were conducted in the same geographical areas and included all children available at that time point. There was no difference in recruitment or follow-up plan.

2. Please provide additional details on why the specific urban slum areas in Vellore were chosen, and discuss any potential implications for generalisability.

The said geographical area was chosen as this urban slum was supported by Low Cost Effective Care Unit (LCECU) of CMC that takes care of urban poor in Vellore. Already we have included the setting as one of the limitations of the study (page 14).

7 Please specify the process of obtaining informed consent, particularly emphasising how consent was obtained from caregivers and assent from children at 9 years.

Thank you. Details are provided in Page 7

“Informed consent for each stage, approved by the IRB had an information sheet about the study and participants were free to choose all or some components of the study – for example, parent could decide about either one or all 9-year assessments namely growth assessment, child cognition assessments, blood tests and MRI brain neuroimaging. Parent gave written signed

consent in the presence of study psychologist who took the consent in the presence of a witness, who also signed the consent form. Additional verbal assent was obtained from the child before each test."

8.Please provide additional information on the adaptation of MISIC from WISC, ensuring clarity on modifications made for cultural relevance.

Thank you. The MISIC manual provides details and adaptations include but not limited to culturally appropriate pictures and settings. Changes made in page 7

9 In terms of data management and statistical analysis- Please Specify if there were any specific procedures or considerations during data management, especially since it was done by the Data Coordinating Centre at the Fogarty International Centre.

Only original data coordination was done by Fogarty International Centre as the original MAL-ED study was conducted in 8 countries. CMC Vellore site has its own data centre which co-ordinated with Fogarty initially, but manages data independently now.

10. Please provide more context for the choice of Pearson correlation. For example, if any assumptions of the Pearson correlation were checked or if there were any concerns regarding the non-normality of continuous variables.

The main variables that were used in these analyses are normally distributed (as expected) and therefore Pearson correlation has been used

11 Please mention the version of STATA software.

It is version STATA version 14 (which is mentioned in the statistical analysis section)

12 Can you pls clarify clarify if there were any significant differences between those who dropped out or didn't have complete data and those who were included in the analysis.

There was no significant difference in sociodemographic characteristics that were observed between children who were followed-up in the cohort to those who dropped out. This has been presented in multiple publications from this MAL-ED India cohort

Our paper "Koshy B, Srinivasan M, Gopalakrishnan S, et al. Are early childhood stunting and catch-up growth associated with school age cognition?-Evidence from an Indian birth cohort. *PloS one* 2022;17(3):e0264010. doi: 10.1371/journal.pone.0264010 [published Online First: 20220302]" addresses this question and shows that there was no difference in the follow-up cohort. This is highlighted in results

13 Can you please expand and provide more explicit, simple and easy to understand guidance on the clinical implications of your findings. How might these results influence early childhood developmental assessments in clinical practice or research especially in LMIC settings? Thank you.

Thank you. Please refer to the response of comment 3 for clarification.

Reviewer: 3

The authors do not adequately justify the necessity of addressing 'stability' in the introduction, and they fall short in providing a solid foundation for employing ICC as an indicator of stability. ICC is commonly utilized to assess inter-rater agreement, reflecting the extent of shared variance among various measures. However, in the context of longitudinal data, the interpretation of common variance can be multifaceted, and the authors do not explicitly articulate the criteria for defining robust common variance. It is crucial to substantiate the use of ICC as a metric for 'stability' in a longitudinal design. Moreover, the introduction should prominently address the rationale behind their interest in stability, as this is of paramount importance. The central concern remains the scientific relevance of documenting stability. If the rationale for this documentation is not clearly articulated, determining an appropriate methodology becomes a challenging task.

Thank you. We have added further text to introduction to highlight the need for stability.

Yes, we agree with the reviewer that ICC is not enough. We also used in addition simple regression and CCC based on the LIN formula.

Reviewer: 4

Thank you for this valuable study exploring the stability of the Bayley Scales and predictability to later cognitive outcomes in childhood in low- middle- income settings. The research question is interesting and worthy of investigation. The manuscript is of high quality, well organised and well written. The clear and transparent description of the study population, study design and methodology and presentation of the results is commended.

Thank you for your encouraging words

I make just a few minor typographical notes and a suggestion for discussion.
1. The authors switch between “BSID” and “Bayley” in text so I suggest reviewing for consistency

Thank you. We have consistently used BSID through the text now

3. Page 10 of 30, line 53 – is this meant to read as "more stable"?

Thank you. Corresponding changes made

3. Page 14 of 30, line- 48 – The BSID scores at 3 years had better predictability of 3- 5- and 7-year cognition – is this meant to read as 5- 7- and 9- year cognition?

Thank you – corresponding changes made.

4. Finally, the authors note in the discussion there has been concerns raised regarding the overestimation of the child development in the BSID-III. The authors may like to consider noting that the revised Bayley-4 has sought to address this.

Thank you – corresponding changes made – page 13/14

VERSION 2 - REVIEW

Reviewer **2**

Name **Bhurawala, Habib**

Affiliation **The University of Sydney Nepean Clinical School, Paediatric Department**

Date **21-Oct-2024**

COI

Many thanks for the opportunity to review this revised manuscript. I am happy with the changes/amendments made by the authors in response to the reviewer comments so not further changes recommended.

Reviewer **3**

Name **Varela , Natalia**

Affiliation

Date **08-Oct-2024**

COI

Thank you for revising comments. Congrats on your study!
